# Anti-Obesity Activity of the Marine Carotenoid Fucoxanthin

**DOI:** 10.3390/md13042196

**Published:** 2015-04-13

**Authors:** Maria Alessandra Gammone, Nicolantonio D’Orazio

**Affiliations:** Human and Clinical Nutrition Unit, Department of Medical, Oral and Biotechnological Sciences, Via Dei Vestini, University G. D’Annunzio, 66013 Chieti, Italy; E-Mail: ndorazio@unich.it

**Keywords:** fucoxanthin, metabolism, obesity, nutrition, functional food

## Abstract

Nowadays the global tendency towards physical activity reduction and an augmented dietary intake of fats, sugars and calories is leading to a growing propagation of overweight, obesity and lifestyle-related diseases, such diabetes, hypertension, dyslipidemia and metabolic syndrome. In particular, obesity, characterized as a state of low-level inflammation, is a powerful determinant both in the development of insulin resistance and in the progression to type 2 diabetes. A few molecular targets offer hope for anti-obesity therapeutics. One of the keys to success could be the induction of uncoupling protein 1 (UCP1) in abdominal white adipose tissue (WAT) and the regulation of cytokine secretions from both abdominal adipose cells and macrophage cells infiltrated into adipose tissue. Anti-obesity effects of fucoxanthin, a characteristic carotenoid, exactly belonging to xanthophylls, have been reported. Nutrigenomic studies reveal that fucoxanthin induces UCP1 in abdominal WAT mitochondria, leading to the oxidation of fatty acids and heat production in WAT. Fucoxanthin improves insulin resistance and decreases blood glucose levels through the regulation of cytokine secretions from WAT. The key structure of anti-obesity effect is suggested to be the carotenoid end of the polyene chromophore, which contains an allenic bond and two hydroxyl groups. Fucoxanthin, which can be isolated from edible brown seaweeds, recently displayed its many physiological functions and biological properties. We reviewed recent studies and this article aims to explain essential background of fucoxanthin, focusing on its promising potential anti-obesity effects. In this respect, fucoxanthin can be developed into promising marine drugs and nutritional products, in order to become a helpful functional food.

## 1. Introduction

Modern lifestyle, characterized by high intakes of fats, sugars and calories as well as a decreased exercise and physical activity, contributes to metabolic and inflammatory diseases, such as obesity, diabetes, hypertension, cancer and other chronic pathologies. Nutrition can play an important role in order to prevent these lifestyle-related disorders, and it is desirable to find safe and effective functional ingredients in food [1]. The importance of marine algae as sources of functional ingredients has been well recognized due to their valuable health beneficial effects. Therefore, isolation and investigation of novel bioactive ingredients with biological activities from marine algae have recently attracted great attention. Among functional ingredients identified from marine algae, fucoxanthin received particular interest. Researchers are focusing on functional ingredients in foods for both prevention and treatment of lifestyle-related diseases. In these respects, marine bioactives, such as marine carotenoids, especially fucoxanthin, are recently gaining attention. Fucoxanthin is a marine carotenoid, which can be found in both macroalgae, such as *Undaria pinnatifida* or *Laminaria japonica*, and microalgae such as *Phaeodactylum tricornutum* or *Cylindrotheca closterium* [2]. Fucoxanthin showed anti-obesity, anti-diabetic, anti-oxidant, anti-inflammatory and anticancer activities [3,4,5,6,7]. Considering the unique structure of fucoxanthin, its metabolism, its safety, as well as its significant bioactivities and pharmacological effects, it can develop as a promising nutritional ingredient and a potential medicinal constituent for human health.

## 2. Structure and Metabolism of Fucoxanthin

**Figure 1:**
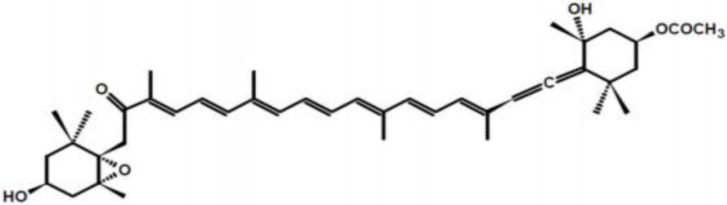
The chemical structure of fucoxanthin.

**Figure 2:**
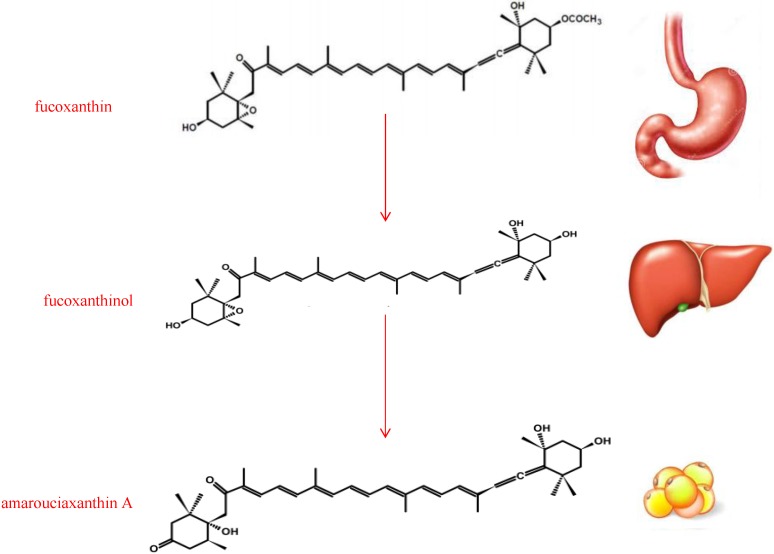
The metabolic conversion of fucoxanthin to amarouciaxanthin A via fucoxanthinol.

Carotenoids, a group of phytochemical substances responsible for the color of some food, play an important role both in the prevention of human diseases and the maintenance of good health. They are classified, according to their source into marine and terrestrial, according to their chemical structure, into carotenes and xanthophylls. Carotenes contain no oxygen, are fat-soluble and insoluble in water (in contrast with other carotenoids, the xanthophylls, which contain oxygen and thus are less chemically hydrophobic); carotenes include beta-carotene and lycopene. Xanthophylls are yellow pigments whose name is due to their formation of the yellow band seen in early chromatography of leaf pigments. Their molecular structure is similar to carotenes, but xanthophylls contain oxygen atoms, while carotenes are purely hydrocarbons with no oxygen. Xanthophylls contain their oxygen either as hydroxyl groups or as pairs of hydrogen atoms that are substituted by oxygen atoms acting as a bridge (epoxide). For this reason, they are more polar than the purely hydrocarbon carotenes, and it is this difference that allows their separations from carotenes in many types of chromatography. Typically, carotenes are more orange in color than xanthophylls. The group of xanthophylls includes (among many other compounds) fucoxanthin, lutein, zeaxanthin, neoxanthin, canthaxanthin, violaxanthin, capsorubin, astaxanthin and α- and β-cryptoxanthin [8], which is the only known xanthophyll containing a beta-ionone ring; thus β-cryptoxanthin is the only xanthophyll that is known to possess pro-vitamin A activity for mammals. In species other than mammals, certain xanthophylls may be converted to hydroxylated retinal-analogues that function directly in vision. They have potential antioxidant biological properties because of their chemical structure and interaction with biological membranes. Their antioxidant properties have been considered the main mechanism by which they afford their beneficial health effects [9]. However, it would be reductive to explain the physiological effects of carotenoids solely by their antioxidant activity. In general, carotenoid plasmatic concentrations reflect concentrations contained in ingested food. Fucoxanthin (Figure 1) is a xanthophyll, whose distinct structure includes an unusual allenic bond, epoxide group, and conjugated carbonyl group in polyene chain with antioxidant properties [10]. Dietary fucoxanthin undergoes metabolic conversion to amarouciaxanthin A via fucoxanthinol in mice [11] requiring Nicotinamide Adenine Dinucleotide Phosphate (NADP+) as cofactor [12], it is hydrolyzed to fucoxanthinol in the gastrointestinal tract by digestive enzymes, such as lipase and cholesterol esterase, and converted to amarouciaxanthin A (Figure 2) in the liver [13]. The bioconversion of fucoxanthinol into amarouciaxanthin A through dehydrogenation/isomerization was principally shown in liver microsomes. The percentage of fucoxanthin, fucoxanthinol, and amarouciaxanthin A in the adipose tissue was 13%, 32%, and 55%, respectively, while the percentage in the other tissues, such as liver, lungs, kidney, heart, and spleen, was 1%–11%, 63%–76% and 20%–26%, respectively, indicating that amarouciaxanthin A accumulated preferentially in the adipose tissue, while fucoxanthinol accumulated mainly in the other tissues [14]. Sangeetha [11] reported various metabolites of fucoxanthin besides the major metabolites fucoxanthinol and amarouciaxanthin A in rats, proposed a possible metabolic pathway of fucoxanthin in the plasma and liver of rats, and speculated that these metabolites might be formed as a result of enzymatic reactions such as isomerization, dehydrogenation, deacetylation, oxidation, and demethylation. Thus, fucoxanthin’s metabolites are considered to be the active forms exerting physiological functions in the body [15]. Amarouciaxanthin A is stored in abdominal white adipose tissue (WAT); fucoxanthinol enters the blood stream and is stored in erythrocytes, liver, lung, kidney, heart, spleen and adipose tissue. Fucoxanthin absorption rate is generally influenced by the composition of food matrix: for example, its solubility in soybean oil and in other vegetable oils is very low, while fucoxanthin can easily dissolve in medium-chain triacylglycerols (MCT) or in fish oil [16].

## 3. Anti-Obesity Effect

**Figure 3:**
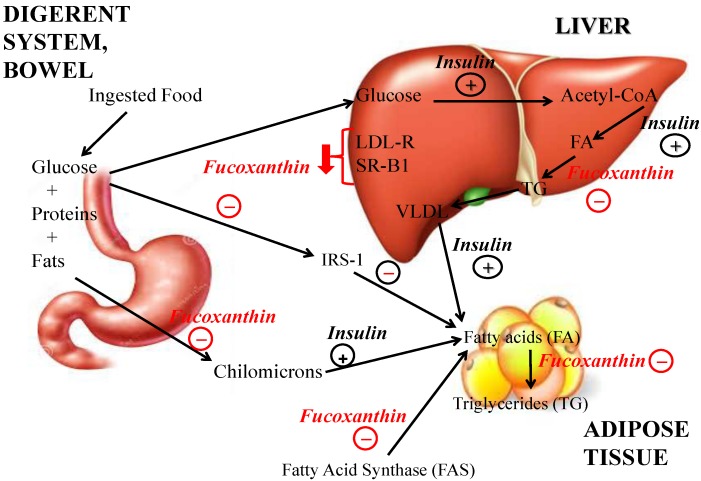
Effects of fucoxanthin on weight loss and lipid metabolism, compared to insulin: fucoxanthin significantly reduces plasmatic and hepatic triglyceride concentrations and cholesterol uptake in the liver via down-regulation of Low-Density Lipoprotein (LDL)-receptor and Scavenger receptor class B member 1 (SR-B1). Fucoxanthin supplementation also decreased mRNA expression of fatty acid synthase (FAS), which catalyzes fatty acid synthesis. It also inhibited the uptake of glucose in mature adipocytes by reducing the phosphorylation of insulin receptor substrate 1 (IRS-1). Fucoxanthin significantly reduced plasmatic and hepatic triglyceride concentrations and positively influenced cholesterol-regulating enzymes activities such as 3-hydroxy-3-methylglutaryl-CoA reductase and acyl-CoA and affects gene expression associated with lipid metabolism: in rats its supplementation decreased mRNA expression of hepatic Acetyl-CoA carboxylase (ACC), a biotin-dependent enzyme that catalyzes the irreversible carboxylation of acetyl-CoA to produce malonyl-CoA.

Long-term unbalanced diets alter lipid metabolism and leads to the accumulation of visceral fat, thus resulting in overweight, obesity and related metabolic disorders, such as diabetes mellitus, hypertension, dyslipidemia and cardiovascular disease. Consequently, a crucial endpoint consists in finding efficient strategies to prevent obesity. In this sense, fucoxanthin can play an anti-obesity effect through several mechanisms. Fucoxanthin significantly reduces plasmatic and hepatic triglyceride concentrations (Figure 3) and positively influenced cholesterol-regulating enzymes such as 3-hydroxy-3-methylglutaryl coenzyme A reductase and acyl-coenzyme A [17]. Fucoxanthin beneficially affects gene expression associated with lipid metabolism: in rats its supplementation decreased mRNA expression of hepatic Acetyl-CoA carboxylase (ACC), a biotin-dependent enzyme that catalyzes the irreversible carboxylation of acetyl-CoA to produce malonyl-CoA. The function of ACC is to up-regulate the metabolism of fatty acids: when the enzyme is active malonyl-CoA is produced. This represents a building block for new fatty acids production and inhibits fatty acyl group transfer from acyl-CoA to carnitine with carnitine-acyltransferase, which inhibits the beta-oxidation of fatty acids in the mitochondria. ACC1 is found in the cytoplasm of all the cells but is more represented in lipogenic tissue, such as adipose tissue and lactating mammary glands, where fatty acid synthesis is particularly important [18]. Fucoxanthin increased High-Density Lipoprotein (HDL)-cholesterol and non-HDL-cholesterol levels in KK-Ay mice (a type 2 diabetic knock-out mouse model that exhibits marked obesity, glucose intolerance, severe insulin resistance, dyslipidemia, and hypertension) by inducing Sterol Regulatory Element-Binding Protein (SREBP) expression and reduced cholesterol uptake in the liver, via down-regulation of Low-Density Lipoprotein (LDL)-receptor and (Scavenger Receptor class B member 1) SR-B1. In fact, hepatic levels of LDL-R and SR-B1 proteins which are important factors for LDL-cholesterol and HDL-cholesterol uptake in the liver from serum, respectively decreased to 60% and 80% in the fucoxanthin-fed mice. Further, dietary fucoxanthin significantly increased the mRNA expression of protein convertase subtilisin/kexin type 9 (PCSK9), which enhances intracellular degradation of LDL-R in lysosomes [19]. Fucoxanthin supplementation also decreased mRNA expression of fatty acid synthase (FAS), a multi-enzyme protein that catalyzes fatty acid synthesis: its main function is to catalyze the synthesis of palmitate from acetyl-CoA and malonyl-CoA into long-chain saturated fatty acids. FAS has been investigated as a possible oncogene, indicator of poor prognosis and a chemotherapeutic target, but it may also be involved in the production of an endogenous ligand of the nuclear receptor PPAR-α, the target of the fibrate drugs administered against hyperlipidemia. Consequently it is currently being investigated as a possible drug target for treating the metabolic syndrome [20]. Recently, stearoyl-coenzyme A desaturase-1 (SCD1) down-regulation has been implicated in the prevention of obesity and in the improvement of insulin and leptin sensitivity. Furthermore, serum leptin levels were significantly decreased in hyperleptinemia KK-A(y) mice after 2 weeks of fucoxanthin feeding, although the suppressive effects of fucoxanthin on hepatic SCD1 and body weight gain were not observed in ob/ob mice [21].

Fucoxanthin also increases the levels of enzyme glucose-6-phosphate dehydrogenase (G6PDH), which is in the pentose-phosphate pathway which supplies energy to cells by maintaining the level of the co-enzyme nicotinamide adenine dinucleotide phosphate (NADPH). This helps maintain appropriate cellular levels of glutathione, thus protecting against oxidative damage [22]. In addition, fucoxanthin also influenced hydroxy-3-methylglutaryl-coenzyme A (HMG-CoA), Acyl-CoA cholesterol acyltransferase (ACAT), as well as sterol regulatory element-binding transcription factors (SREBP-1): SREBP-1a, whose activity is regulated by sterol levels in the cell, regulates genes related to lipid and cholesterol production [23], and SREBP-1C, which regulates genes required for both glucidic metabolism and fatty acid/lipid production; its expression is modulated by insulin [24]. Even mRNA expression of lecithin-cholesterol acyltransferase (LCAT), an enzyme that converts free cholesterol into cholesteryl ester, and carnitine palmitoyl-transferase (CPT1) were significantly increased after fucoxanthin administration. Neo-synthesized cholesteryl ester is then sequestered into the core of a lipoprotein particle, making the newly synthesized HDL [25]. CPT1 is a mitochondrial enzyme responsible for the formation of acyl carnitines by catalyzing the transfer of the acyl group of a long-chain fatty acyl-CoA from coenzyme A to l-carnitine, thus resulting an essential step in the beta-oxidation of long chain fatty acids. Three isoforms of CPT1 are currently known: CPT1A which is present in liver, CPT1B in muscle and CPT1C in brain. This enzyme can be inhibited by malonyl-CoA, the first intermediate produced during fatty acid synthesis. Its role in fatty acid metabolism makes CPT1 important in many metabolic disorders, such as diabetes and insulin resistance. In these metabolic diseases, free fatty acid (FFA) levels get elevated, adipose tissue accumulate in skeletal muscle and the ability of muscles to oxidize fatty acids slowly decreases. The increased levels of malonyl-CoA caused by hyperglycemia and hyperinsulinemia, inhibit CPT1; CPT1 inhibition causes a subsequent decrease in the transport of long chain fatty acids into muscle and heart mitochondria, thus decreasing fatty acid oxidation in such cells. The shunting of LCFAs away from mitochondria leads to the observed increase in FFA levels and the accumulation of fat in skeletal muscle. In this respect, CPT1 up-regulation by fucoxanthin plays a critical role in preventing and limiting these symptoms [26]. Maeda *et al.* reported that increased mRNA expression of monocyte chemoattractant protein-1 (MCP-1) was observed in high fat (HF) mice, but was normalized in the fucoxanthin-rich lipids (FLs) group: the HF-FL diet could suppress high fat (HF) diet-induced obesity in mice [27]. Woo *et al.* [4] also discovered that mRNA expressions of acyl-coA oxidase1, palmitoyl (ACOX1) and peroxisome proliferators activated receptor α (PPARα) and γ (PPARγ), which is an important modulator for UCP1 expression [28], were obviously modified by fucoxanthin. Recent studies showed that pre-adipocyte differentiation is divided into early (days 0–2, D0–D2), intermediate (days 2–4, D2–D4), and late stages (day 4 onwards, D4) [29]. Fucoxanthin presents different effects on cells during the three differentiation stages: during early differentiation stages (D0–D2), fucoxanthin promoted adipocyte differentiation and increased protein expression of PPARγ, CCAAT/enhancer-binding protein α (C/EBPα), sterol regulatory element-binding protein 1c (SREBP1c) and adiponectin mRNA expression. However, fucoxanthin inhibits intercellular lipid accumulation by reducing the expression of PPARγ, C/EBPα, and SREBP1c during the intermediate (D2–D4) and late stages (D4–D7) of differentiation [30]. In addition, the metabolite of fucoxanthin, fucoxanthinol, was reported to down-regulate PPARγ and exhibited stronger suppressive effects than fucoxanthin on adipocyte differentiation [31]. Amarouciaxanthin A, another metabolite of fucoxanthin was also showed to suppress PPARγ and C/EBPα expression during adipocyte differentiation. Compared to fucoxanthinol, amarouciaxanthin A markedly down-regulated the mRNA expression of adipocyte fatty acid binding protein (aP2), lipoprotein lipase (LPL), and glucose-transporter 4 (Glut-4) [32]. Therefore, at the molecular level, fucoxanthin increased protein expression of PPARγ, C/EBPα, SREBP1c, aP2 and adiponectin mRNA expression in a dose-dependent manner in early stage of adipocyte differentiation, while it reduced the expression of PPARγ, C/EBPα, and SREBP1c during the intermediate and late differentiation stages. It also inhibited the uptake of glucose in mature adipocytes (Figure 3) by reducing the phosphorylation of insulin receptor substrate 1 (IRS-1). These results suggest that fucoxanthin exerts differing effects on different differentiation stages and inhibits glucose uptake in mature adipocytes [30].

### 3.1. Fucoxanthin and Uncoupling Proteins: Adaptive Thermogenesis as a Physiological Defense against Obesity

**Figure 4:**
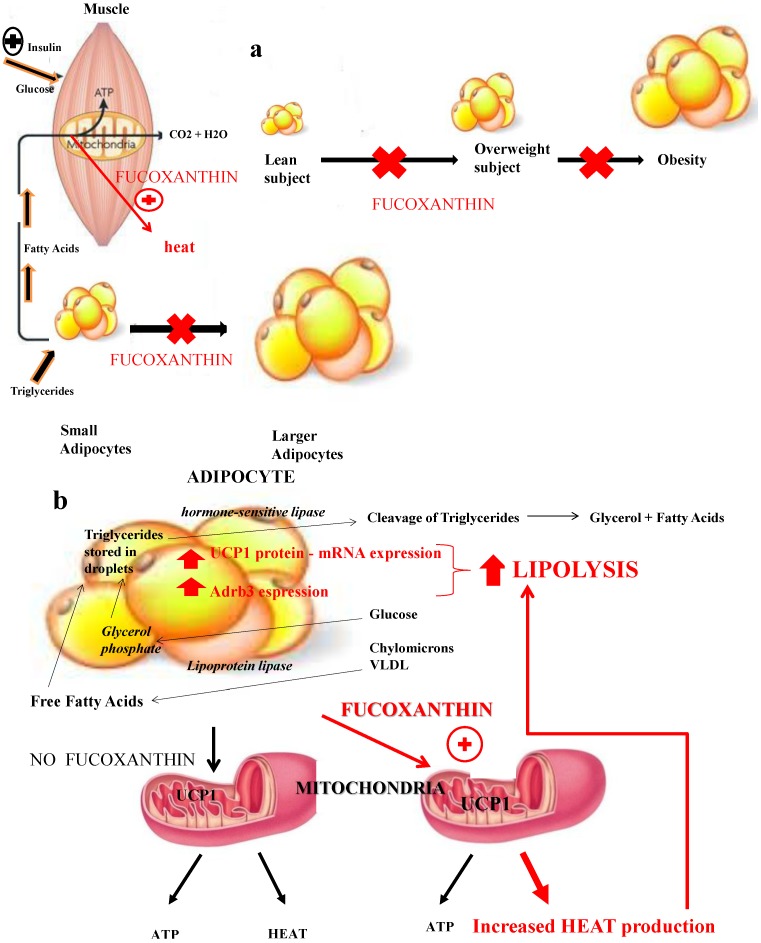
Effects of fucoxanthin on thermogenesis and lipolysis: the muscle (**a**) and the adipose tissue (**b**). Fucoxanthin plays an anti-obesity effect mainly by stimulating uncoupling protein-1 (UCP-1) expression in white adipose tissue (WAT). This protein, situated in the mitochondrial inner cellular membrane, is usually found in brown adipose tissue (BAT) and it is not expressed in WAT in absence of any stimulation. Physiologic bodily metabolism determines heat production: this process is named thermogenesis and UCP-1 dissipates the pH-gradient generated by oxidative phosphorylation, releasing chemical energy as heat. Fucoxanthin was found to promote not only UCP1 protein and mRNA expression but also β3-adrenergic receptor (Adrb3), which is responsible for lipolysis and thermogenesis. This increased sensitivity to sympathetic nerve stimulation may lead to a further up-regulation of fat oxidation in WAT. This adaptive thermogenesis plays a crucial role in energy expenditure as heat, in order to limit weight gain and to favor weight loss.

Recent literature [33,34] suggests that fucoxanthin plays an anti-obesity effect, mainly by stimulating uncoupling protein-1 (UCP-1) expression in white adipose tissue (WAT). This protein, situated in the mitochondrial inner cellular membrane, is usually found in brown adipose tissue (BAT) and it is not expressed in WAT in absence of any stimulation. Physiologic bodily metabolism determines heat production: this process is named thermogenesis and UCP-1 dissipates the pH-gradient generated by oxidative phosphorylation, through releasing chemical energy as heat. *UCP1* gene expression, which is stimulated by many factors, such as cold, β3-agonists, adrenergic stimulation and thyroid hormones, represents a significant part of body energy expenditure, its dysfunction resulting an important cause of weight gain and a significant cofactor for the development of obesity [29]. Fucoxanthin augments the amount of energy, which is released as heat in fat tissue, thus stimulating thermogenesis. UCP-1 and mRNA could be detected in WAT when experimental animals received *Undaria* lipids containing fucoxanthin: 0.2% fucoxanthin in their diet significantly attenuated weight gain in mice, by increasing UCP-1 expression [35]. This UCP1 induction also in white adipose tissue (WAT) by fucoxanthin and its derivatives leads to fatty acids oxidation and heat production in WAT [30]. This adaptive thermogenesis plays a crucial role in energy expenditure as heat, in order to limit weight gain and to favor weight loss. Fucoxanthin was found to promote not only UCP1 protein and mRNA expression in WAT of obese animals but also β3-adrenergic receptor (Adrb3), which is responsible for lipolysis and thermogenesis (Figure 4) [35]. This increased sensitivity to sympathetic nerve stimulation may lead to a further up-regulation of fat oxidation in WAT. Fucoxanthin supplementation was also tested in humans for weight loss: a 16-week supplementation with 4.0 mg/day showed an important increase in resting energy expenditure (REE), which was measured by indirect calorimetry. This augmented REE resulted to be even more important at a dose of 8 mg [36]. A significant reduction in body weight and fat in obese individuals resulted in the down-regulation of inflammatory and hepatic markers such as C-reactive protein (CRP), glutamic pyruvic transaminase (GPT), glutamic oxaloacetic transaminase (GOT), γ-glutamyl transpeptidase (γGT), thus preventing metabolic syndrome [37]. Furthermore, it was suggested that fucoxanthin improved insulin resistance and decreased blood glucose level, at least in part, through down-regulating adipokines, tumor necrosis factor-α, monocyte chemoattractant protein-1, interleukin-6, and plasminogen activator inhibitor-1, because it down-regulates their mRNA expression by directly acting on adipocytes and macrophages in white adipose tissue. In addition, fucoxanthin acts through an up-regulation of glucose transporter 4 in skeletal muscle in KK-Ay mice [38].

### 3.2. Fucoxanthin and Leptin Regulation

The hormone leptin is primarily expressed in differentiated adipocytes of white fat tissue and plays a crucial role in maintaining the homeostatic control of adipose tissue and body weight, by regulating food intake and energy expenditure through several neural and endocrine mechanisms. In obesity the expression of the leptin gene and its circulating concentration are elevated without any regulatory effects on body weight, with the development of resistance [39]. After its discovery leptin became the great hope as an anti-obesity treatment because of its ability to reduce food intake and increase energy expenditure. However, treating obese people with exogenous leptin resulted mostly unsuccessful since they present already high circulating leptin levels to which they do not respond anymore: this situation defines the state of leptin resistance [40]. Fucoxanthin might alter plasma leptin level in order to achieve the anti-obesity action. Many previous studies reported that leptin secretions are elevated due to the accumulation of fat in adipocytes and leptin could control body weight and adipose fat pad through the regulation of the energy expenditure: in particular, Park *et al.* [41] performed a study evaluating the beneficial effect of *Undaria pinnatifida* ethanol extract in C57BL/6J mice and found fucoxanthin could significantly decrease plasma leptin level and that was associated with a significant decrement of the epididymal adipose tissue weight. In this study, fucoxanthin supplement remarkably reduced the adipocyte size compared to the control group. Fasting blood glucose, plasma leptin and insulin levels were significantly higher in control group by 1.5–2.3-folds. Fucoxanthin significantly lowered blood glucose of 19.8% and insulin of about 33%, compared to control. The plasma leptin concentration showed a positive correlation with body weight and resulted to get lower after fucoxanthin supplementation. Another relevant study displayed that fucoxanthin down-regulates stearoyl-coenzyme A desaturase-1 (SCD1), with subsequent improvement of insulin and leptin sensitivity, thus contributing to the prevention of obesity. In fact, SCD1 is a rate-limiting enzyme that catalyzes the biosynthesis of monounsaturated fatty acids from saturated fatty acids. A diet containing 0.2% fucoxanthin for 2 weeks in obese mouse models of hyperleptinemia KK-A(y) markedly suppressed SCD1 mRNA and protein expressions in the liver. Furthermore, serum leptin levels were significantly decreased in hyperleptinemia KK-A(y) mice after 2 weeks of fucoxanthin feeding, although the suppressive effects of fucoxanthin on hepatic SCD1 and body weight gain were not observed in ob/ob mice. These results show that fucoxanthin down-regulates SCD1 expression and alters fatty acid composition of the liver via regulation of leptin signaling in hyperleptinemia KK-A(y) mice but not in leptin-deficient ob/ob mice [21]. The fatty acid composition of liver lipids was also affected by an observed decrease in the ratio of oleic acid to stearic acid. In these animals with leptin resistance, a leptin decrease (that should lead to an increase in food intake in normal animals) has beneficial effects. These results show that fucoxanthin down-regulates SCD1 expression and alters fatty acid composition of the liver via regulation of leptin signaling in hyperleptinemia KK-A y mice but not in leptin-deficient ob/ob mice. Not only leptin, but also orexin and ghrelin are implicated in regulation of energy homeostasis. The levels of these substances exhibit circadian fluctuations, and abnormalities in these rhythms were observed in obesity and metabolic syndrome. On the one hand, serum leptin levels increase during night, while cerebrospinal fluid orexin levels augment during active phase; on the other hand, plasma ghrelin concentrations were increased before meals and during night. High concentrations of leptin during sleep help keeping sleep by inhibition of feeding behavior and arousal through inhibition of neuropeptide Y and orexin neurons, while high concentrations of ghrelin before meal might enhance wakefulness through activation of orexin neurons [42]. These recent findings suggest that the circadian rhythms of these substances are important for the maintenance of basal metabolism and of normal energy homeostasis. The discovery that orexin neurons are regulated by peripheral metabolic cues, including ghrelin and leptin and that they might have important roles as a link between the coordination of feeding and sleep/wakefulness states, suggests roles of orexin in the maintenance of energy homeostasis [43]. In addition, basal metabolism and energy expenditure also influence appetite control, feeding and energy intake: this dynamic interaction makes difficult the prediction of a resultant shift in energy balance, and therefore weight change. It is well recognized that the major influences on hunger and subsequent weight control arise from fat-free mass and fat mass, resting metabolic rate, gastric adjustment to ingested food, changes in episodic and circadian peptides such as insulin, ghrelin, cholecystokinin, glucagon-like peptide-1 and leptin [44]. The study of potential interference of fucoxanthin with these peptides and other neuropeptides regulating food intake and metabolism could open new horizons: the specific actions of fucoxanthin on each physiological component, as well as the eventual interaction with food composition represent an interesting target for future research.

## 4. Obesity and Non Alcoholic Fat Liver Disease: Hepatoprotective Effect of Fucoxanthin

**Figure 5:**
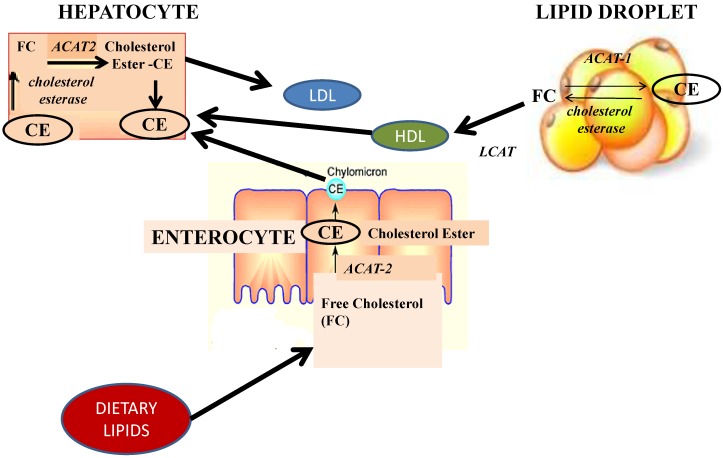
Effects of fucoxanthin on glucidic and lipid metabolism: from the enterocyte to the hepatocyte. Fucoxanthin increases fatty acids oxidation and decreases the hepatic lipid contents by regulating metabolic enzyme activities and stimulating β-oxidation activity. Hepatic lipid contents resulted to be markedly lower after fucoxanthin supplementation because fucoxanthin inhibits hepatic lipogenic enzymes, glucose-6-phosphate dehydrogenase, malic enzyme, fatty acid synthase and phosphatidate phosphohydrolase, which are involved in the hepatic lipid droplet. In addition, an important effect of fucoxanthin in enterocytes, such as competition with lipid absorption can be postulated.

Increased fatty acid oxidation contributes to improve fatty liver by decreasing the amounts of fatty acids as substrates for triacylglycerol synthesis [37]. Fucoxanthin increases fatty acid oxidation and decreases the hepatic lipid contents by regulating metabolic enzyme activities and stimulating β-oxidation activity [4]. In fact, hepatic lipid contents resulted to be markedly lower after fucoxanthin supplementation compared to the control group, because fucoxanthin inhibits hepatic lipogenic enzymes, glucose-6-phosphate dehydrogenase, malic enzyme, fatty acid synthase and phosphatidate phosphohydrolase, which are involved in the hepatic lipid droplet (Figure 5); in addition fucoxanthin stimulates β-oxidation activity. Fucoxanthin and its metabolite fucoxanthinol were reported to promote the proportion of docosahexaenoic acid (DHA) in mice’s liver [45,46,47]. Fucoxanthin significantly up-regulated glycolytic enzyme such as glucokinase in the liver, and thus increased the ratio of hepatic glucokinase/glucose-6-phosphatase and glycogen content, indicating that fucoxanthin normalized the hepatic glycogen content in high-fat fed mice [41]. The reduction of liver lipids might be due to the increase of docosahexaenoic acid, which reduces the activity of hepatic enzymes in fatty acid synthesis and increases hepatic fatty acid β-oxidation, in the liver [45]. Fucoxanthin and fucoxanthinol enhanced the amount of docosahexaenoic acid in the liver of KK-Ay mice, whereas the level of docosahexaenoic acid in the small intestine remained unaltered. In addition, an increase in arachidonic acid was also found in fucoxanthin-fed mice, indicating that fucoxanthin might modify the metabolic pathways of ω-3 and ω-6 highly unsaturated fatty acids [46]. Recently, Airanthi *et al.* [48] showed that the levels of docosahexaenoic acid and arachidonic acid in liver lipids of KK-Ay mice given the lipids from brown seaweeds significantly increased. Recent pharmacological studies in Non Alcoholic Fat Liver Disease (NAFLD) animal models and in adult humans, which demonstrate DHA both anti-inflammatory and insulin sensitizing properties, suggested a potential role of fucoxanthin in treatment of NAFLD [49].

## 5. Obesity and Oxidative Stress: Antioxidant and Anti-Inflammatory Effects of Fucoxanthin

A high fat diet can cause obesity, and obesity causes overproduction of reactive oxygen species (ROS) [50] which are responsible for cellular damage. Fucoxanthin chemical structure contains an epoxide group and hydroxyl group which are considered to be strong antioxidants [10], thus its supplementation could reduce oxidative stress [51]. *In vivo* experiments determined some markers of antioxidant capacity, such as plasma total antioxidant capacity (TAC), antioxidant enzymes such as catalase, superoxide dismutase (SOD), and glutathione peroxidase (GSH-Px), mRNA expression of transcription factor such as nuclear erythroid factor like 2 (Nrf2), and its target genes such as NADPH quinone oxidoreductase1 (NQO1). GSH-Px plasmatic activity in rats, which followed a high fat diet with fucoxanthin, was significantly higher compared to the high fat diet group which did not take fucoxanthin. Plasmatic TAC level, mRNA expression of Nrf2 and NQO1, catalase and hepatic GSH-Px activities were also significantly higher in the supplemented group [52].

In order to determine whether fucoxanthin activates cellular antioxidant enzymes via up-regulation of the Nrf2/antioxidant-response element (ARE) pathway, an interesting study was conducted through the incubation of murine hepatic BNL CL.2 cells with fucoxanthin for 24 h. Fucoxanthin (≥5 μM) resulted to increase ROS at 6 h of incubation, whereas pre-incubation with α-tocopherol (30 μM) decreased the increase of ROS, thus indicating the pro-oxidant nature of fucoxanthin. Fucoxanthin significantly enhanced extracellular signal-regulated kinases (ERK) and p38 phosphorylation and markedly increased nuclear factor erythroid-derived 2-like 2 (Nrf2) protein accumulation after incubation for 12 h. Moreover, fucoxanthin significantly augmented binding activities of nuclear Nrf2 with ARE and increased mRNA and protein expression of heme oxygenase-1 (HO-1), an enzyme that is induced in response to stress, and NAD(P)H dehydrogenase quinone-1 (NQO1) after incubation for 12 h. Thus, fucoxanthin may exert its antioxidant activity, at least partly, through its pro-oxidant actions [53]. The MAPK/ERK pathway is a chain of proteins in the cell that communicates a signal from a receptor on the surface of the cell to the DNA in the nucleus of the cell. When one of the proteins in the pathway is mutated, it can become stuck in the “on” or “off” position, which is a necessary step in the development of many cancers. Components of the MAPK/ERK pathway were discovered when they were found in cancer cells. Drugs that reverse this switch are being investigated as cancer treatments [54]. In particular, fucoxanthin evidenced antitumor activity in human leukemia cell HL-60 cells via the induction of apoptosis [55]: this study focused on the effect of fucoxanthin induction on the accumulation of ROS and on the triggering of Bcl-xL signaling pathway in HL-60 cells, determining that ROS are generated during fucoxanthin-induced cytotoxicity and apoptosis in HL-60 cells, and that *N*-acetylcysteine (NAC), a ROS scavenger, inhibited fucoxanthin-induced cytotoxicity and apoptosis. In this study, it was displayed that fucoxanthin generated ROS and that the accumulation of ROS performed a fundamental role in the fucoxanthin-induced Bcl-xL signaling pathway.

Kim *et al.* [56] investigated the anti-inflammatory effects of fucoxanthin in lipopolysaccharide (LPS)-stimulated murine macrophages, displaying that fucoxanthin could reduce the levels of pro-inflammatory mediators such as NO, PGE_2_, IL-1β, TNF-α, and IL-6 by suppressing the NF-κB activation and the MAPK phosphorylation. These anti-inflammatory activities of fucoxanthin were demonstrated *in vivo* too [57]: fucoxanthin markedly inhibited the antigen-induced release of β-hexosaminidase in both rat basophilic leukemia 2H3 cells and mouse bone marrow-derived mast cells; in addition it suppressed antigen induced aggregation of the high affinity IgE receptor, which is crucial in regulating signals of mast cells.

## 6. Genetic and Iatrogenic Aspects of Obesity: The Potential of Fucoxanthin

Genetic and iatrogenic factors play an important role in the development of lifestyle-related diseases, such as type 2 diabetes mellitus (DM2) and obesity, and it is well established that genetically susceptible subjects can develop these metabolic diseases after being exposed to environmental risk factors. In this respect, great efforts have been recently made to identify genes associated with overweight and obesity. UCP1 is mainly expressed in brown adipose tissue, and acts in thermogenesis, regulation of energy expenditure, and protection against oxidative stress, like discussed above: all these mechanisms are associated with the pathogenesis not only of obesity but also of DM2. Hence, *UCP1* gene is implicated in the development of these disorders: several studies have reported that polymorphisms -*3826A/G*, -*1766A/G* and -*112A/C* in its promoter region, *Ala64Thr* in exon 2 and *Met299Leu* in exon 5 are possibly associated with obesity [58]. Similar to UCP1, the β3-adrenergic receptor gene (β3-AR) is expressed in BAT and WAT and plays an important role in the induction of lipolysis and in the regulation of energy homeostasis. In addition, it is the main adrenoreceptor that stimulates UCP1 expression. The *Trp64Arg* polymorphism in the β3-AR gene has been associated with weight gain and other obesity-related indexes, as well as with insulin resistance in different populations [59]. Interestingly, literature shows that a synergistic effect between the -*3826A/G* polymorphism of *UCP1* gene and the *Trp64Arg* polymorphism of β3-AR gene is associated with an increased tendency for weight gain [60], resistance to weight loss, or subsequent weight-maintenance after a low-calorie diet [61]. In contrast, some studies did not display any influence of the interaction between these two polymorphisms on the resistance to a low-calorie diet, BMI and other obesity-related metabolic parameters [62]. Ethnical and age differences, as well as environmental factors and a synergistic effect with other genes could explain these controversial findings among different investigations. Obesity can be caused by a variety of factors: environmental, genetic, personal, and often, medical factors. An important drugs side effect is represented by weight gain and even iatrogenic obesity: a number of medications can promote weight gain or hinder weight loss, and weight-neutral alternatives are not often available. Some possibly adipogenic medications are represented by psychoactive drugs, such as antipsychotics and antidepressant. Weight gain is a well-documented side effect of antipsychotic treatment. It is generally not proportional to dose, and can vary from minimum weight gains over several years to enormous ones over just a few months. Both typical and novel antipsychotics are associated with weight gain, but novel antipsychotics, especially clozapine and olanzapine, have the greatest adipogenic potential and carry the greatest risk for the development of hypertension, diabetes and lipid abnormalities. While discontinuing antipsychotic medications in patients who suffer from psychosis results not recommendable, these medications are increasingly used off-label as mood stabilizers and sleep aids. Second-generation antipsychotics have been associated with an increased liability for weight gain and metabolic side effects. Among them, clozapine and olanzapine showed great liability to induce weight gain and metabolic adverse reactions. A recent study found that the uses of olanzapine and clozapine were associated with changes in leptin, adipocytokines and total ghrelin: olanzapine had greater influences on adiponectin and total ghrelin than clozapine. The changes in adipocytokines and total ghrelin were a direct effect of antipsychotics on hormonal pathways of energy homeostasis, rather than the result of weight gain [63]: in this respect, the potential interference of fucoxanthin with UCP-1 and with these peptides and other neuropeptides regulating food intake and metabolism could result helpful during antipsychotic treatment in order to limit the subsequent weight gain. Also tricyclic antidepressants are often associated with weight gain. Amitriptyline appears to have the greatest obesogenic potential [64]. Reduced energy expenditure appears to be behind the weight-promoting effect of these drugs, while changes in food intake contribute to a smaller extent. Weight gain, generally tied to increased food intake, is also common with lithium treatment (where it appears to be dose-related, and is more likely to occur in women who are already overweight) and with antiepileptic drugs, in particular valproate and carbamazepine [65]. A number of other medications have been found to cause weight gain and favor obesity in patients: steroids, which increase abdominal adiposity and insulin resistance, thus heightening risk for diabetes and cardiovascular disease; antihistamines [64], which often make patients lethargic, so that they find it more difficult to exercise; antibiotics, which affect the microbiota in the intestinal system. In fact, accumulating evidence [66,67] indicates that the gut microbiota plays a significant role in the development of obesity, obesity-associated inflammation and insulin resistance. How the microbiota promotes human health and disease is a rich area of investigation that is likely to generate fundamental discoveries in energy metabolism and may lead to new strategies for prevention of obesity and its complications. Therefore, several drugs are associated with weight change of varying magnitude, this consciousness should guide the choice of drug when several options exist and promote the institution of preemptive strategies for weight management, such as fucoxanthin supplementation, when drugs with known weight effects are prescribed.

## 7. Conclusions

Obesity is a multifactorial disease associated with both genetic and environmental factors. Knowledge on factors associated with these disorders allow us to better understand them, and may provide more effective approaches to treatment and prevention. In this respect, fucoxanthin has many bioactivities potentially promoting human health. In particular, its potential anti-obesity effect was primarily detected by murine studies, which displayed an induction of uncoupling protein-1 in abdominal white adipose tissue mitochondria, leading to the oxidation of fatty acids and heat production. Even if further studies are necessary in order to authenticate these results in humans too, all the promising scientific findings might allow its future development as a marine nutraceutical and an interesting functional food. However, it is well recognized that basal metabolism, appetite and food intake are affected by many other factors including age, sex, body composition, physical activity levels, environmental factors (such as temperature), and also individual differences. These factors are likely to influence the balance of homeostatic controls and make it difficult to generalize; nevertheless, research and knowledge advancement in this area brings hope that potential novel strategies for weight control could be on the horizon.

## References

[1] Kuipers R.S., de Graaf D.J., Luxwolda M.F., Muskiet M.H., Dijck-Brouwer D.A., Muskiet F.A. (2011). Saturated fat, carbohydrates and cardiovascular disease. Neth. J. Med..

[2] Kim S.M., Jung Y.H., Kwon O., Cha K.H., Um B.H. (2012). A potential commercial source of fucoxanthin extracted from the microalga *Phaeodactylum tricornutum*. Appl. Biochem. Biotechnol..

[3] Maeda H., Hosokawa M., Sashima T., Miyashita K. (2007). Dietary combination of fucoxanthin and fish oil attenuates the weight gain of white adipose tissue and decreases blood glucose in obese/diabetic KK-Ay mice. J. Agric. Food Chem..

[4] Woo M.N., Jeon S.M., Kim H.J., Lee M.K., Shin S.K., Shin Y.C., Park Y.B., Choi M.S. (2010). Fucoxanthin supplementation improves plasma and hepatic lipid metabolism and blood glucose concentration in high-fat fed C57BL/6N mice. Chem. Biol. Interact..

[5] Sangeetha R.K., Bhaskar N., Baskaran V. (2009). Comparative effects of β-carotene and fucoxanthin on retinol deficiency induced oxidative stress in rats. Mol. Cell. Biochem..

[6] Lee S.J., Bai S.K., Lee K.S., Namkoong S., Na H.J., Ha K.S., Han J.A., Yim S.V., Chang K., Kwon Y.G. (2003). Astaxanthin inhibits nitric oxide production and inflammatory gene expression by suppressing I(κ)B kinase-dependent NF-κB activation. Mol. Cells.

[7] Kim K.N., Ahn G., Heo S.J., Kang S.M., Kang M.C., Yang H.M., Kim D., Roh S.W., Kim S.K., Jeon B.T. (2013). Inhibition of tumor growth *in vitro* and *in vivo* by fucoxanthin against melanoma B16-F10 cells. Envir. Toxicol. Pharmacol..

[8] McNulty H., Jacob R.F., Mason R.P. (2008). Biologic activity of carotenoids related to distinct membrane physicochemical interactions. Am. J. Cardiol..

[9] Seifried H.E., Anderson D.E., Fisher E.I., Milner J.A. (2007). A review of the interaction among dietary antioxidants and reactive oxygen species. J. Nutr. Biochem..

[10] Hu T., Liu D., Chen Y., Wu J., Wang S. (2010). Antioxidant activity of sulfated polysaccharide fractions extracted from *Undaria pinnitafida in vitro*. Int. J. Biol. Macromol..

[11] Sangeetha R.K., Bhaskar N., Divakar S., Baskaran V. (2010). Bioavailability and metabolism of fucoxanthin in rats: Structural characterization of metabolites by LC–MS (APCI). Mol. Cell. Biochem..

[12] Das S.K., Hashimoto T., Kanazawa K. (2008). Growth inhibition of human hepatic carcinoma Hep-G2 cells by fucoxanthin is associated with down-regulation of cyclin D. Biochim. Biophys. Acta.

[13] Asai A., Sugawara T., Ono H., Nagao A. (2004). Biotransformation of fucoxanthinol in amarouciaxanthin A in mice and Hep-G2 cells: Formation and cytotoxicity of fucoxanthin metabolites. Drug Metab. Dispos..

[14] Hashimoto T., Ozaki Y., Taminato M., Das S.K., Mizuno M., Yoshimura K., Maoka T., Kanazawa K. (2009). The distribution and accumulation of fucoxanthin and its metabolites after oral administration in mice. Br. J. Nutr..

[15] Gammone M.A., Riccioni G., D’Orazio N. (2015). Carotenoids: Potential allies of cardiovascular health?. Food Nutr. Res..

[16] Asai A., Yonekura L., Nagao A. (2008). Low bioavailability of dietary epoxy-xanthophylls in humans. Br. J. Nutr..

[17] Hu X., Li Y., Li C., Fu Y., Cai F., Chen Q., Li D. (2012). Combination of fucoxanthin and conjugated linoleic acid attenuates body weight gain and improves lipid metabolism in high-fat diet-induced obese rats. Arch. Biochem. Biophys..

[18] Tong L. (2005). Acetyl-coenzyme A carboxylase: Crucial metabolic enzyme and attractive target for drug discovery. Cell. Mol. Life Sci..

[19] Beppu F., Hosokawa M., Niwano Y., Miyashita K. (2012). Effects of dietary fucoxanthin on cholesterol metabolism in diabetic/obese KK-A(y) mice. Lipids Health Dis..

[20] Wu M., Singh S.B., Wang J., Chung C.C., Salituro G., Karanam B.V., Lee S.H., Powles M., Ellsworth K.P., Lassman M.E. (2011). Antidiabetic and antisteatotic effects of the selective fatty acid synthase (FAS) inhibitor platensimycin in mouse models of diabetes. Proc. Natl. Acad. Sci. USA.

[21] Beppu F., Hosokawa M., Yim M.J., Shinoda T., Miyashita K. (2013). Down-regulation of hepatic stearoyl-CoA desaturase-1 expression by fucoxanthin via leptin signaling in diabetic/obese KK-A(y) mice. Lipids.

[22] Aster J., Kumar V., Robbins S.L., Abbas A.K., Fausto N., Cotran R.S. (2010). Robbins and Cotran Pathologic Basis of Disease.

[23] Eberlé D., Hegarty B., Bossard P., Ferré P., Foufelle F. (2004). SREBP transcription factors: Master regulators of lipid homeostasis. Biochimie.

[24] Ferré P., Foufelle F. (2010). Hepatic steatosis: A role for *de novo* lipogenesis and the transcription factor SREBP-1c. Diabetes Obes. Metab..

[25] DeVries R., Borggreve S.E., Dullaart R.P. (2004). Role of lipases, lecithin: Cholesterol acyltransferase and cholesteryl ester transfer protein in abnormal high density lipoprotein metabolism in insulin resistance and type 2 diabetes mellitus. Clin. Lab..

[26] Rasmussen B.B., Holmbäck U.C., Volpi E., Morio-Liondore B., Paddon-Jones D., Wolfe R.R. (2002). Malonyl coenzyme A and the regulation of functional carnitine palmitoyltransferase-1 activity and fat oxidation in human skeletal muscle. J. Clin. Invest..

[27] Maeda H., Hosokawa M., Sashima T., Murakami-Funayama K., Miyashita K. (2009). Anti-obesity and anti-diabetic effects of fucoxanthin on diet-induced obesity conditionsin a murine model. Mol. Med. Rep..

[28] Kang S.I., Shin H.S., Kim H.M., Yoon S.A., Kang S.W., Kim J.H., Ko H.C., Kim S.J. (2012). Petalonia binghamiae extract and its constituent fucoxanthin ameliorate high-fat diet-induced obesity by activating AMP-activated protein kinase. J. Agric. Food Chem..

[29] Ntambi J.M., Kim Y.C. (2000). Adipocyte differentiation and gene expression. J. Nutr..

[30] Kang S.I., Ko H.C., Shin H.S., Kim H.M., Hong Y.S., Lee N.H., Kim S.J. (2011). Fucoxanthin exerts differing effects on 3T3-L1 cells according to differentiation stage and inhibits glucose uptake in mature adipocytes. Biochem. Biophys. Res. Commun..

[31] Maeda H., Hosokawa M., Sashima T., Takahashi N., Kawada T., Miyashita K. (2006). Fucoxanthin and its metabolite, fucoxanthinol, suppress adipocyte differentiation in 3T3-L1 cells. Intern. J. Mol. Med..

[32] Yim M.J., Hosokawa M., Mizushina Y., Yoshida H., Saito Y., Miyashita K. (2011). Suppressive effects of amarouciaxanthin A on 3T3-L1 adipocyte differentiation through down-regulation of PPAR-γ and C/EBPr mRNA expression. J. Agric. Food Chem..

[33] D’Orazio N., Gemello E., Gammone M.A., DeGirolamo M., Ficoneri C., Riccioni G. (2012). Fucoxantin: A treasure from the sea. Mar. Drugs.

[34] Gammone M.A., Gemello E., Riccioni G., D’Orazio N. (2014). Marine bioactives and potential application in sports. Mar. Drugs.

[35] Maeda H., Hosokawa M., Sashima T., Funayama K., Miyashita K. (2005). Fucoxanthin from edible seaweed *Undaria pinnatifida*, shows anti-obesity effect through UCP1 expression in white adipose tissues. Biochem. Biophys. Res. Commun..

[36] Abidov M., Ramazanov Z., Seifulla R., Grachev S. (2010). The effects of Xanthigen in the weight management of obese premenopausal women with non-alcoholic fatty liver disease and normal liver fat. Diabet. Obes. Metable.

[37] Heilbronn L.K., Noakes M., Clifton M.P. (2001). Energy restriction and weight loss on very-low-fat diets reduce C-reactive protein concentrations in obese, healthy women. Atheroscler. Thromb. Vasc. Biol..

[38] Hosokawa M., Miyashita T., Nishikawa S., Emi S., Tsukui T., Beppu F., Okada T., Miyashita K. (2010). Fucoxanthin regulates adipocytokine mRNA expression in white adipose tissue of diabetic/obese KK-Ay mice. Arch. Biochem. Biophys..

[39] Gautron L., Elmquist J.K. (2011). Sixteen years and counting: An update on leptin in energy balance. J. Clin. Invest..

[40] Roujeau C., Jockers R., Dam J. (2014). New pharmacological perspectives for the leptin receptor in the treatment of obesity. Front Endocrinol..

[41] Park H.J., Lee M.K., Park Y.B., Shin Y.C., Choi M.S. (2011). Beneficial effects of *Undaria pinnatifida* ethanol extract on diet-induced-insulin resistance in C57BL/6J mice. Food Chem. Toxicol..

[42] Tsujino N., Sakurai T. (2012). Circadian rhythm of leptin, orexin and ghrelin. Nihon Rinsho.

[43] Sakurai T. (2006). Roles of orexins and orexin receptors in central regulation of feeding behavior and energy homeostasis. CNS Neurol. Disord. Drug Targets.

[44] Blundell J.E., Gibbons C., Caudwell P., Finlayson G., Hopkins M. (2015). Appetite control and energy balance: Impact of exercise. Obes. Rev..

[45] Maeda H., Tsukui T., Sashima T., Hosokawa M., Miyashita K. (2008). Seaweed carotenoid, fucoxanthin, as a multi-functional nutrient. Asia Pac. J. Clin. Nutr..

[46] Tsukui T., Konno K., Hosokawa M., Maeda H., Sashima T., Miyashita K. (2007). Fucoxanthin and fucoxanthinol enhance the amount of docosahexaenoic acid in the liver of KKAy obese/diabetic mice. J. Agric. Food Chem..

[47] Tsukui T., Baba T., Hosokawa M., Sashima T., Miyashita K. (2009). Enhancement of hepatic docosahexaenoic acid and arachidonic acid contents in C57BL/6J mice by dietary fucoxanthin. Fish. Sci..

[48] Airanthi M.K.W.A., Sasaki N., Iwasaki S., Baba N., Abe M., Hosokawa M., Miyashita K. (2011). Effect of brown seaweed lipids on fatty acid composition and lipid hydroperoxide levels of mouse liver. J. Agric. Food Chem..

[49] Masterton G.S., Plevris J.N., Hayes P.C. (2010). Review article: Omega-3 fatty acids–A promising novel therapy for non-alcoholic fatty liver disease. Aliment. Pharmacol. Ther..

[50] Dandona P., Aljada A., Chaudhuri A., Mohanty P., Garg R. (2005). Metabolic syndrome: A comprehensive perspective based on interactions between obesity, diabetes, and inflammation. Circulation.

[51] D’Orazio N., Gammone M.A., Gemello E., DeGirolamo M., Cusenza S., Riccioni G. (2012). Marine bioactives: Pharmacological properties and potential applications against inflammatory diseases. Mar. Drugs.

[52] Ha A.W., Na S.J., Kim W.K. (2013). Antioxidant effects of fucoxanthin rich powder in rats fed with high fat diet. Nutr. Res. Pract..

[53] Liu C.L., Chiu Y.T., Hu M.L. (2011). Fucoxanthin enhances HO-1 and NQO1 expression in murine hepatic BNL CL.2 cells through activation of the Nrf2/ARE system partially by its pro-oxidant activity. J. Agric. Food Chem..

[54] Orton R.J., Sturm O.E., Vyshemirsky V., Calder M., Gilbert D.R., Kolch W. (2005). Computational modelling of the receptor-tyrosine-kinase-activated MAPK pathway. Biochem. J..

[55] Kim K.N., Heo S.J., Kang S.M., Ahn G., Jeon Y.J. (2010). Fucoxanthin induces apoptosis in human leukemia HL-60 cells through a ROS-mediated Bcl-xL pathway. Toxicol. Vitro.

[56] Kim K.N., Heo S.J., Yoon W.J., Kang S.M., Ahn G., Yi T.H., Jeon Y.J. (2010). Fucoxanthin inhibits the inflammatory response by suppressing the activation of NF-κB and MAPKs in lipopolysaccharide-induced RAW 264.7 macrophages. Eur. J. Pharmacol..

[57] Sakai S., Sugawara T., Matsubara K., Hirata T. (2009). Inhibitory effect of carotenoids on the degranulation of mast cells via suppression of antigen-induced aggregation of high affinity IgE receptor. J. Biol. Chem..

[58] Brondani L.A., Assmann T.S., Duarte G.C., Gross J.L., Canani L.H., Crispim D. (2012). The role of the uncoupling protein 1 (UCP1) on the development of obesity and type 2 diabetes mellitus. Arq Bras. Endocrinol. Metabol..

[59] Jia J.J., Tian Y.B., Cao Z.H., Tao L.L., Zhang X., Gao S.Z. (2010). The polymorphisms of UCP1 genes associated with fat metabolism, obesity and diabetes. Mol. Biol. Rep..

[60] Clément K., Ruiz J., Cassard-Doulcier A.M., Bouillaud F., Ricquier D., Basdevant A., Guy-Grand B., Froguel P. (1996). Additive effect of A→G (-3826) variant of the uncoupling protein gene and the Trp64Arg mutation of the β3-adrenergic receptor gene on weight gain in morbid obesity. Int. J. Obes. Relat. Metab. Disord..

[61] Fogelholm M., Valve R., Kukkonen-Harjula K., Nenonen A., Hakkarainen V., Laakso M. (1998). Additive effects of the mutations in the β3-adrenergic receptor and uncoupling protein-1 genes on weight loss and weight maintenance in Finnish women. J. Clin. Endocrinol. Metab..

[62] Schäffler A., Palitzsch K.D., Watzlawek E., Drobnik W., Schwer H., Schölmerich J. (1999). Frequency and significance of the A→G (-3826) polymorphism in the promoter of the gene for uncoupling protein-1 with regard to metabolic parameters and adipocyte transcription factor binding in a large population-based Caucasian cohort. Eur. J. Clin. Invest..

[63] Lu M., Wang T., Lin T., Shao W., Chang S., Chou J., Ho Y., Liao Y., Chen V.C. (2014). Differential effects of olanzapine and clozapine on plasma levels of adipocytokines and total ghrelin. Prog. Neuropsychopharmacol. Biol. Psychiatry.

[64] Domecq J.P., Prutsky G., Leppin A., Sonbol M.B., Altayar O., Undavalli C., Wang Z., Elraiyah T., Brito J.P., Mauck K.F. (2015). Drugs commonly associated with weight change: A systematic review and meta-analysis. J. Clin. Endocrinol. Metab..

[65] Lamp Y., Eshel Y., Rapaport A., Sarova-Pinhas I. (1991). Weight gain, increased appetite, and excessive food intake induced by carbamazepine. Clin. Neuropharmacol..

[66] Shen J., Obin M.S., Zhao L. (2013). The gut microbiota, obesity and insulin resistance. Mol. Aspects Med..

[67] Stachowicz N., Kiersztan A. (2013). The role of gut microbiota in the pathogenesis of obesity and diabetes. Postepy Hig. Med. Dosw..

